# Monocarboxylate transporter 1 contributes to growth factor-induced tumor cell migration independent of transporter activity

**DOI:** 10.18632/oncotarget.9016

**Published:** 2016-04-26

**Authors:** Alana L. Gray, David T. Coleman, Runhua Shi, James A. Cardelli

**Affiliations:** ^1^ Louisiana State University Health Sciences Center–Shreveport, Shreveport, LA, USA; ^2^ Feist-Weiller Cancer Center, Shreveport, LA, USA

**Keywords:** MCT1, HGF, EGF, cancer, motility

## Abstract

Tumor progression to metastatic disease contributes to the vast majority of incurable cancer. Understanding the processes leading to advanced stage cancer is important for the development of future therapeutic strategies. Here, we establish a connection between tumor cell migration, a prerequisite to metastasis, and monocarboxylate transporter 1 (MCT1). MCT1 transporter activity is known to regulate aspects of tumor progression and, as such, is a clinically relevant target for treating cancer. Knockdown of MCT1 expression caused decreased hepatocyte growth factor (HGF)-induced as well as epidermal growth factor (EGF)-induced tumor cell scattering and wound healing. Western blot analysis suggested that MCT1 knockdown (KD) hinders signaling through the HGF receptor (c-Met) but not the EGF receptor. Exogenous, membrane-permeable MCT1 substrates were not able to rescue motility in MCT1 KD cells, nor was pharmacologic inhibition of MCT1 able to recapitulate decreased cell motility as seen with MCT1 KD cells, indicating transporter activity of MCT1 was dispensable for EGF- and HGF-induced motility. These results indicate MCT1 expression, independent of transporter activity, is required for growth factor-induced tumor cell motility. The findings presented herein suggest a novel function for MCT1 in tumor progression independent of its role as a monocarboxylate transporter.

## INTRODUCTION

Recent data from the Centers for Disease Control show that cancer is the second leading cause of death in the United States [1]. Although benign tumors are typically treatable, locally confined tumor cells can become invasive and motile [2], increasing the likelihood of metastatic tumor formation and decreasing the probability of effective treatment. Therefore, preventing tumor progression to metastatic disease is critical to increasing survival of cancer patients, as supported by the widely reported statistic that metastasis is responsible for > 90% of all cancer-related deaths.

A defined series of molecular and cellular events leading to tumor cell dissemination and establishment of metastatic lesions composes a process known as the metastatic cascade [3]. The acquisition of tumor cell motility and subsequent invasion of the nearby basement membrane are key steps of this cascade. Recent mathematical modeling suggesting motility is critical to tumor growth rates further supports the importance of tumor cell motility in tumor progression [4]. Signaling through receptor tyrosine kinases (RTKs) is a major contributing factor to tumor cell motility and invasiveness, particularly through initiation of an epithelial-to-mesenchymal transition (EMT) [5, 6]. Accumulating evidence implicating RTKs in cancer progression and EMT has led to the approval of multiple tyrosine kinase inhibitors (TKIs) for use in cancer patients [7, 8]. Two highly studied RTK signaling pathways, the epidermal growth factor receptor (EGFR; Entrez Gene: 1956) pathway and the hepatocyte growth factor receptor (HGFR or c-Met; Entrez Gene: 4233) pathway, have been identified as therapeutic targets for preventing and treating metastatic cancer [9–12]. Unfortunately, while these compounds have proven to be highly efficacious in certain patient populations, they are only approved to treat a handful of cancer subtypes [7]. Furthermore, there are multiple reports of TKI resistance, often through kinase crosstalk as well as overabundance of growth factors, like HGF (Entrez Gene: 3082), in the surrounding tumor microenvironment [13–17]. Because of this, continued investigation into the interplay and unexpected influences of signaling networks is warranted for the development of novel efficacious therapeutic strategies.

Through studies examining metabolic contributions to growth factor signaling (data not published), we discovered that monocarboxylate transporters (MCTs) are important factors associated with EGF- and HGF-induced cancer cell motility. MCTs are necessary for the transport of monocarboxylates, such as lactate and pyruvate, as well as ketone bodies and short-chain fatty acids, across the cell membrane [18–20]. There are 14 members of the solute carrier 16A family, but only some of the proton-coupled MCTs (MCTs 1, 2, 4; Entrez Genes: 6566, 9194, 9123) and the sodium-coupled MCT (sMCT1; Entrez Gene: 160728) have been associated with cancer [21–23]. Specifically, MCT1 expression has been correlated with cancer [24–27]. Moreover, the transporting activity of MCT1 has been shown to regulate aspects of tumor progression, such as tumor cell motility and angiogenesis [24, 28, 29].

Here, we attempt to further elucidate the mechanisms regulating growth factor signaling. In particular, we establish a connection between growth factor-induced tumor cell motility and MCT1 expression.

## RESULTS

### DU145 and HCC1806 cells primarily express MCT1 and MCT4

**Figure 1:**
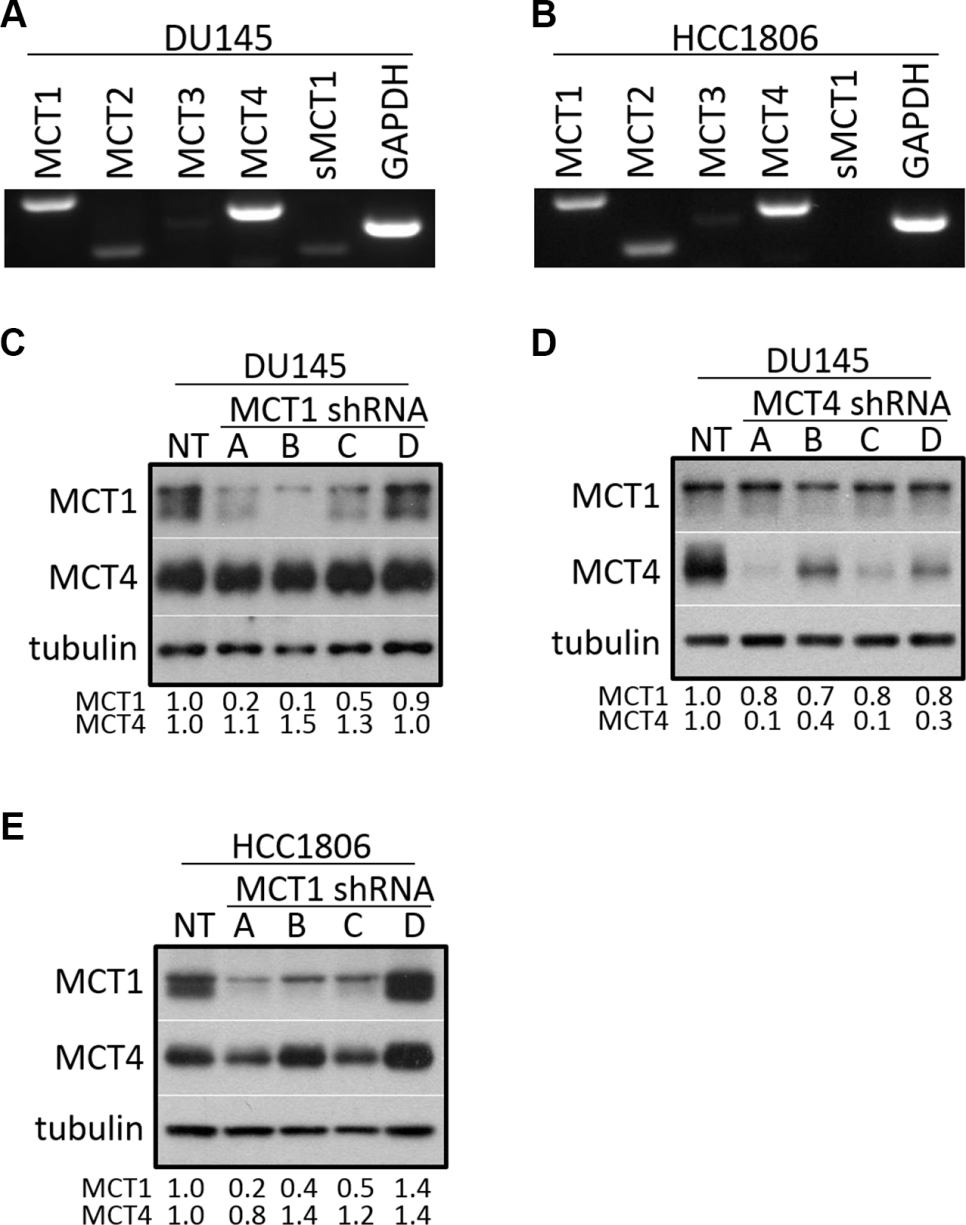
DU145 and HCC1806 cells primarily express MCT1 and MCT4 (**A**–**B**) RNA was extracted from DU145 (A) and HCC1806 (B) cells and subjected to reverse transcriptase-PCR using primers to MCT1, MCT2, MCT3, MCT4, sMCT1, and GAPDH. (**C**–**E**) Cells were transduced with lentiviral-delivered shRNA using four different shRNA sequences targeted to MCT1 (A–D) (C: DU145, E: HCC1806) or MCT4 (A–D) (D: DU145), as well as non-targeted shRNA (NT) and analyzed for the indicated proteins by western blot. Relative expression was quantitated using densitometry.

c-Met signaling has a well-established role in prostate cancer and triple negative breast cancer [30–34]. Endpoint PCR revealed that DU145 prostate cancer cells (Figure 1A) and HCC1806 triple negative breast cancer cells (Figure 1B) predominantly express MCT1 and MCT4 out of all cancer-associated and proton-linked MCTs analyzed. MCT2 and MCT3 (Entrez Gene: 23539) mRNA appears to be present in these cells, but to a lesser degree although MCT2 is expressed almost at similar levels in HCC1806 cells when compared to MCT1. Since MCT1 and MCT4 were consistently expressed higher than the other analyzed MCTs, we chose to focus on the contribution of these two transporters to growth factor signaling.

To study the connection between MCT1 or MCT4 and the EGFR and c-Met signaling axes, lentiviral-delivered shRNA was used to reduce expression of either MCT1 or MCT4 in DU145 cells. Multiple distinct shRNA sequences were used to reduce MCT1 and MCT4 expression, respectively (Figure 1C and 1D). HCC1806 breast cancer cells were also treated with MCT1 shRNA (Figure 1E). Notably, MCT1 knockdown (KD) resulted in minimal effects on MCT4 expression (Figure 1C and 1E) and there was a slight reduction in MCT1 expression in some of the MCT4 KD clones (Figure 1D). For the majority of the presented work, results obtained using MCT1 KD clone A and MCT4 KD clone A are shown; however, all results were confirmed using multiple clones.

### MCT1 KD reduces EGF- and HGF-induced cell motility

**Figure 2:**
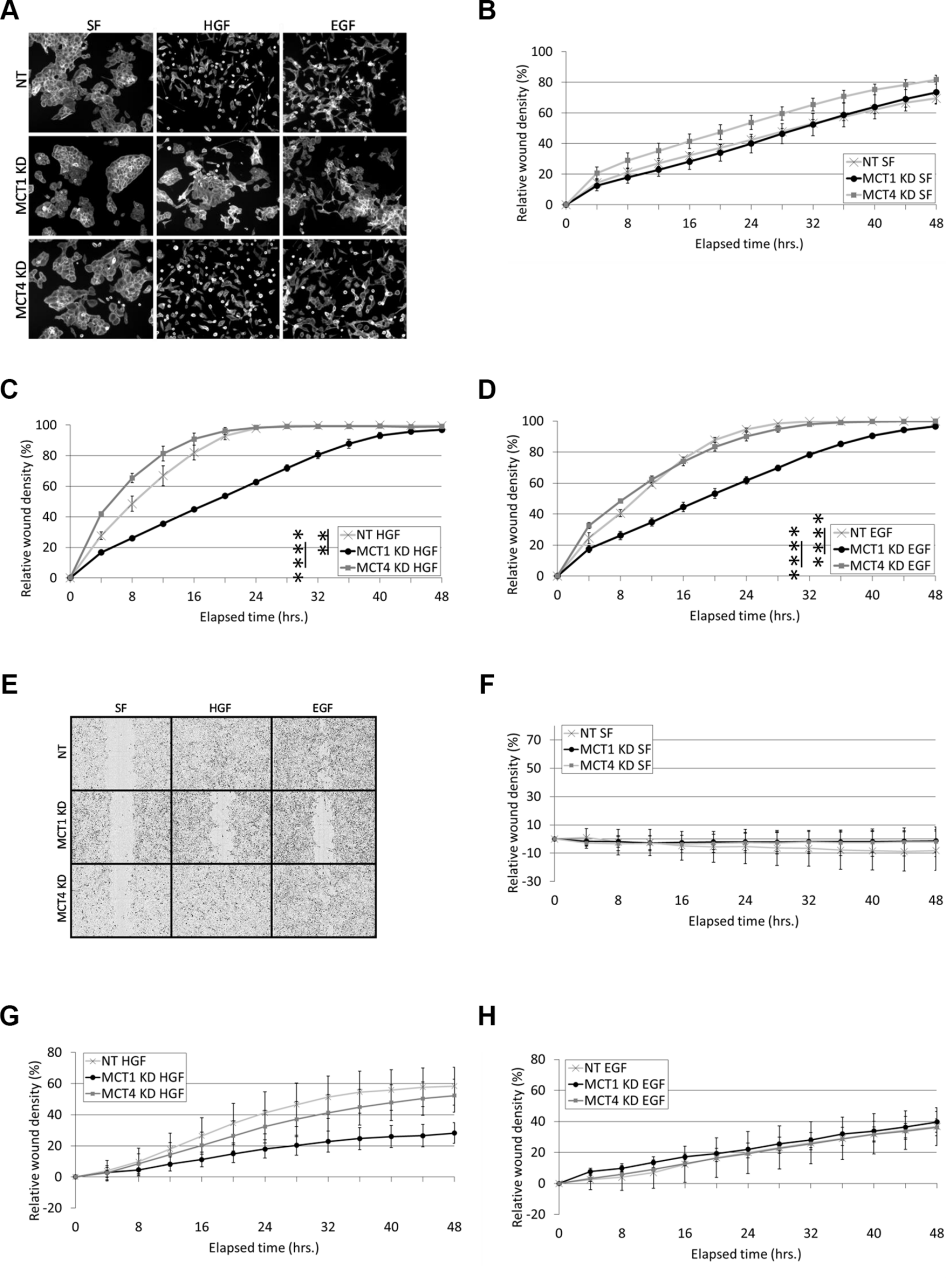
MCT1 KD reduces HGF- and EGF-induced cell motility (**A**) DU145 cells stably expressing NT, MCT1, or MCT4 shRNA were treated in serum-free (SF) media with or without 33 ng/ml HGF or 100 ng/ml EGF overnight. Cells were fixed and stained for actin. Representative 10X images are shown. (**B**–**E**) DU145 NT, MCT1 KD, and MCT4 KD cells were treated with SF media (B), 33 ng/ml HGF (C), or 100 ng/ml EGF (D) for the indicated times. Prior to treatment, confluent monolayers were wounded and washed with complete media. Results were quantitated using IncuCyte™ imaging software; *n* = 3. Representative images of wound healing at the 24 hour timepoint are shown in (E). (**F**–**H**) DU145 NT, MCT1 KD, and MCT4 KD cells were grown to ~100% confluency. Cells were wounded and washed with complete media prior to overlaying with Matrigel diluted 1:5 in SF media. Cells were treated in SF media (F), 33 ng/ml HGF (G), or 100 ng/ml EGF (H) for the indicated times; *n* = 3. Quantitative data represent mean ± SEM. **p < 0.01, ****p < 0.0001.

In response to EGF and HGF, DU145 cells exhibit a motogenic response in the form of cell scattering and collective wound healing. Functional studies using the DU145 MCT1 and MCT4 stable KD cell lines revealed that knockdown of MCT1 prevented cell scattering mediated by overnight treatment with EGF (Entrez Gene: 1950) and HGF, while MCT4 KD cells still scattered (Figure 2A). Similarly, MCT1 KD significantly reduced the rate of EGF- and HGF-induced wound healing when compared to NT cells (Figure 2C and 2D). MCT4 KD had insignificant effects on HGF- and EGF-induced wound healing, such that differences between MCT1 KD vs. MCT4 KD EGF- and HGF-induced wound healing were also significant, similar to MCT1 KD vs. NT cells (Figure 2C and 2D). Representative wound healing images taken at the 24 hour timepoint are shown in Figure 2E.

In a Matrigel^®^-based assay, DU145 cells are not invasive unless stimulated with growth factor (Figure 2F). We found that MCT1 KD did not reduce EGF-induced invasion (Figure 2H), but did slow the rate of HGF-induced invasion, although not significantly (Figure 2G). Notably, comparison of NT and MCT1 KD or MCT4 KD untreated, serum-free (SF) conditions shows that neither MCT1 KD nor MCT4 KD affect general, non-growth factor stimulated cell motility or invasion (Figure 2B and 2F).

### MCT1 KD reduces c-Met expression and HGF-induced c-Met phosphorylation

**Figure 3:**
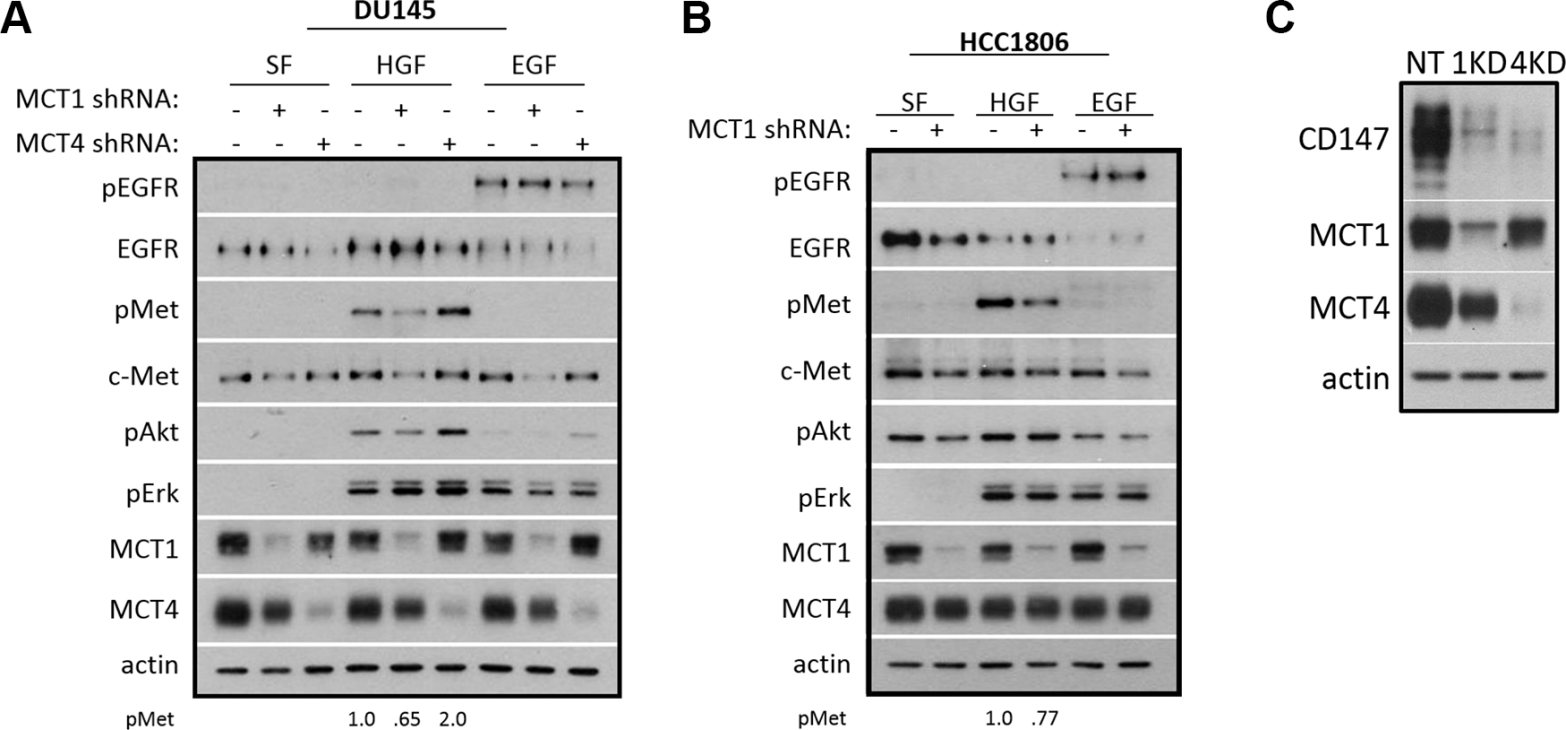
MCT1 KD reduces HGF signaling and c-Met expression (**A**) DU145 NT, MCT1 KD, and MCT4 KD and (**B**) HCC1806 NT and MCT1 KD cells were seeded and grown to ~70% confluency. Cells were treated with or without 33 ng/ml HGF or 100 ng/ml EGF for 20 minutes in serum-free (SF) media after serum-starving 30 minutes. Lysates were collected and analyzed by western blot for the indicated proteins. Densitometric analysis of pMet is shown. (**C**) CD147 expression was analyzed by western blot in DU145 NT, MCT1 KD, and MCT4 KD cell lysates.

The dramatic decrease in EGF- and HGF-induced cell motility in MCT1 KD cells suggested that MCT1 KD may affect c-Met and EGFR signaling pathways. Western blot analysis showed that, indeed, MCT1 KD reduced levels of phosphorylated c-Met (pMet), as well as downstream phosphorylated Akt (pAkt) in DU145 cells exposed to HGF; however, there was no decrease in activation of EGFR in DU145 cells treated with EGF (Figure 3A). The reduction in c-Met activation following HGF treatment was recapitulated in HCC1806 MCT1 KD cells, although consistently no effect on pAkt was observed (Figure 3B). We also found that MCT1 KD resulted in lower levels of total c-Met but not EGFR (Figure 3A and 3B). The decrease in pMet appeared to coincide with the loss of total c-Met, suggesting that the perceived reduction in HGF-mediated activation of c-Met is likely due to the reduction in total c-Met protein.

Further, CD147 (Entrez Gene: 682) has been demonstrated to be a chaperone for MCT1 and MCT4 expression [35]. Initial investigations found CD147 expression to be reduced following knockdown of MCT1, suggesting CD147 might be involved in reduced signaling, c-Met expression, or motility phenotypes in MCT1 KD cells. However, further western blot analysis showed that levels of CD147 were reduced in MCT1 KD cells as well as MCT4 KD cells (Figure 3C). Therefore, since CD147 is also decreased in MCT4 KD cells, decreased CD147 expression is not responsible for the unique phenotypes seen in MCT1 KD cells.

### Neither MCT1 KD nor MCT4 KD affects proliferation or ATP production

**Figure 4:**
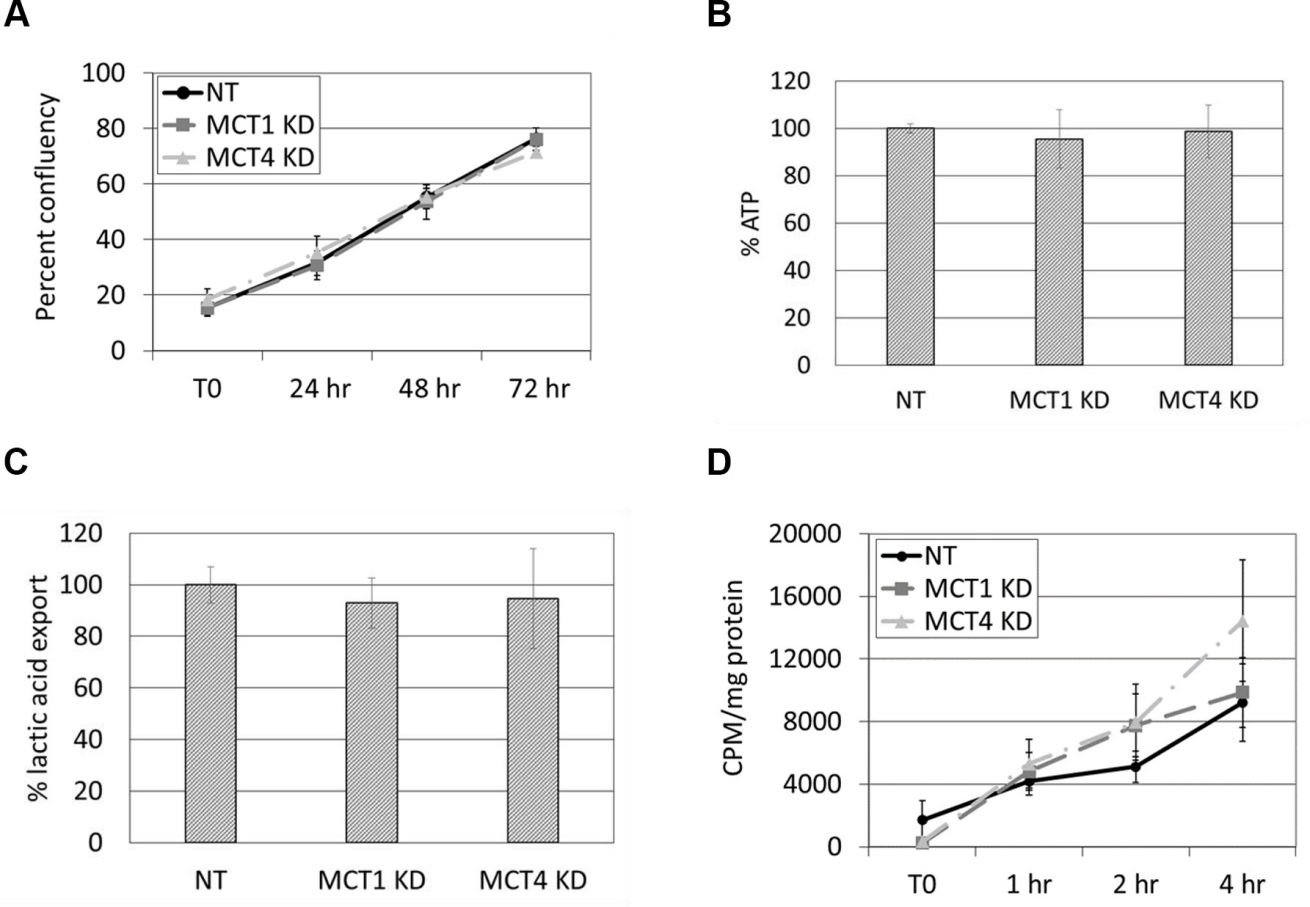
Proliferation, energy production, and overall lactic acid transport are not affected by knockdown of MCT1 or MCT4 (**A**) Proliferation in complete media containing puromycin over 72 hours was measured using an IncuCyte™ imaging system. Data are shown as percent confluency; *n* = 3. (**B**) Cellular ATP levels were measured following 24 hours in serum-free media. Data are shown as percent ATP normalized to NT control cells set at 100%; *n* = 3. (**C**) Serum-free, lactic acid-free media was placed on cells for 24 hours prior to measurement of lactic acid in the supernatant. Data are shown as percent export of lactic acid normalized to NT control cells set at 100%; *n* = 5. (**D**) Cells were labeled with ^14^C-lactic acid in HEPES for the indicated times. Uptake of ^14^C-lactic acid was determined by measuring counts per minute (CPM) normalized to protein; *n* = 4. All data represent mean ± SEM.

To test whether the reduction in cell motility in MCT1 KD cells was due to a reduction in cell growth or energy, proliferation and ATP production were examined in DU145 NT, MCT1 KD, and MCT4 KD cells. Based on automated analysis of cell confluency over time, no significant changes in proliferation were observed between NT and MCT1 KD or MCT4 KD cells (Figure 4A). Similarly, fluorescence-based detection of ATP levels found no difference in NT vs. MCT1 KD or MCT4 KD (Figure 4B). These data suggest that the reduction in motility in MCT1 KD cells cannot be attributed to defects in cell growth or energy production.

### Neither MCT1 KD nor MCT4 KD affects overall uptake or export of lactic acid

Functionally, MCT1 and MCT4 are responsible for the transport of monocarboxylic acids, such as lactic acid, across the cell membrane. MCT1 is commonly responsible for lactic acid import while MCT4 has a greater propensity for lactic acid export, although these transporters can perform either function [36]. We hypothesized that knockdown of MCT1 would result in decreased import and/or export of these substrates, thereby affecting cell motility in response to growth factors. To test this, we measured levels of lactic acid export in NT, MCT1 KD, and MCT4 KD cells (Figure 4C). Following overnight treatment with fresh serum-free, lactic acid-free media, we found that neither the reduction of MCT1 nor the reduction of MCT4 expression affected extrusion of lactic acid from the cell.

Reports in the literature suggest that MCT1 has a stronger affinity for bringing lactic acid into the cell [36, 37], so it was possible that import of lactic acid could be altered in MCT1 KD cells. However, measurement of ^14^C-lactic acid import in NT, MCT1 KD, and MCT4 KD cells showed no change in the rate of lactic acid uptake over the course of several hours (Figure 4D). To ensure that ^14^C-lactic acid uptake was transporter-mediated, we used excess non-radiolabeled lactic acid to compete with the radiolabeled lactic acid and also excess, non-radiolabeled pyruvic acid which is transported by the same MCTs as lactic acid. Both pyruvic acid and lactic acid competed with the ^14^C-lactic acid, as evidenced by reduced uptake of ^14^C-lactic acid (Figure S1A). These data confirmed that, in our system, uptake of lactic acid is carrier mediated and that the absence of a difference between NT vs. MCT1 KD and MCT4 KD cells is not due to non-specific diffusion of ^14^C-lactic acid across the cell membrane. Ultimately, redundancy between MCT1 and MCT4 is likely responsible for the lack of change in overall uptake or export of lactic acid. Collectively, these data indicate that reduced expression of MCT1 negatively affects growth factor-induced cell motility despite unchanged net lactic acid transport.

### MCT1 substrates are not sufficient to rescue motility defects in MCT1 KD cells

To confirm that a decrease in uptake of monocarboxylates is not responsible for decreased growth factor-induced motility, we incubated cells with millimolar concentrations of methyl-lactic acid, a membrane-permeable form of lactic acid, to determine if delivering MCT1 substrates to the intracellular space while bypassing MCT1-mediated transport could rescue defects in EGF- and HGF-induced cell motility. Figure S2B shows that methylated lactic acid (ME-LA) was unable to rescue motility blocked by MCT1 KD. In a similar experiment, methylated membrane-permeable pyruvic acid was also unable to rescue motility (data not shown). These data suggest that the substrates transported by MCT1 are not sufficient for cell motility induced by EGF and HGF and that decreased import of these substrates is not responsible for decreased cell motility in MCT1 KD cells.

### MCT1 inhibition does not affect growth factor-induced cell motility or c-Met signaling

**Figure 5:**
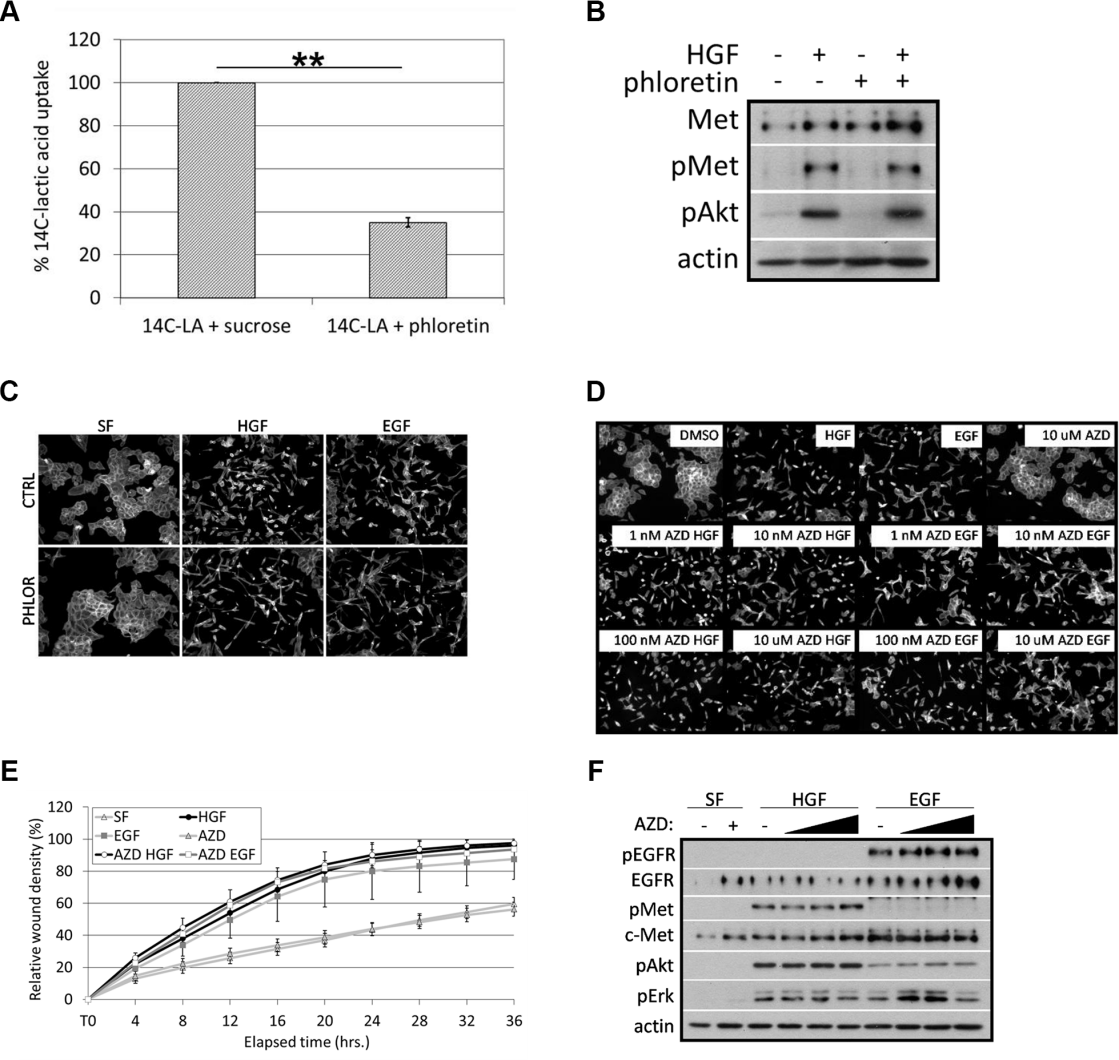
Inhibition of MCT1 activity does not affect HGF- or EGF-induced cell motility or c-Met signaling (**A**) DU145 cells were labeled with ^14^C-lactic acid in the absence (sucrose) or presence of 40 μM phloretin for 1 hour. Data are shown as percent ^14^C-lactic acid uptake normalized to control set at 100%; *n* = 3. (**B**) Cells were treated with 40 μM phloretin with or without 33 ng/ml HGF for 20 minutes. Cell lysates were analyzed by western blot for the indicated proteins. (**C**) DU145 cells were treated with 40 μM phloretin or without (CTRL) in the presence or absence of 33 ng/ml HGF or 100 ng/ml EGF overnight. (**D**) DU145 cells were treated with or without 33 ng/ml HGF or 100 ng/ml EGF overnight in the presence or absence of AZD3965 at 1 nM, 10 nM, 100 nM, or 10 μM. (C, D) Cells were fixed and stained for actin. Representative 10× images are shown. (**E**) Confluent monolayers of DU145 cells were wounded and washed with PBS prior to overnight treatment in serum-free (SF) media with or without 33 ng/ml HGF or 100 ng/ml EGF in the presence or absence of 10 μM AZD3965. Results were quantitated using IncuCyte™ imaging software; *n* = 3. (**F**) DU145 cells were treated with or without 10 nM, 100 nM, or 100 μM AZD3965 in the presence or absence of 33 ng/ml HGF or 100 ng/ml EGF for 20 minutes. Cell lysates were analyzed by western blot for the indicated proteins. Quantitative data represent mean ± SEM. **p < 0.01.

Although we reduced MCT1 expression and likely its activity, the redundant transporter activity of MCT4 has been shown to mask changes in lactic acid transport following MCT1 inhibition, potentially explaining the lack of change in overall uptake and export of lactic acid in MCT1 KD cells [38, 39]. Therefore, in an attempt to determine if reduced transporter activity of MCT1 accounted for defects in motility and signaling in MCT1 KD cells, we treated DU145 wild-type cells with phloretin, a broad inhibitor of MCT1, MCT2, and MCT4 [36]. This general MCT inhibitor limited uptake of ^14^C-lactic acid (Figure 5A); however, at the same concentration, phloretin did not prevent c-Met activation (Figure 5B) or EGF- or HGF-induced cell scattering (Figure 5C). These data began to suggest that MCT1 transporter activity is not required for growth factor-induced motile phenotypes.

To more thoroughly address whether MCT1 activity is required for growth factor-induced signaling and motility, we obtained AZD3965, a specific MCT1 inhibitor. AZD3965 has a reported IC_100_ of 100 nM for MCT1-mediated lactic acid uptake [40]; however, when DU145 wild-type cells were treated with AZD3965 at concentrations 1000-fold higher than the IC_100_, an approximately 50% reduction in ^14^C-lactic acid uptake was observed (Figure S2A). Again, we believe that this was due to the presence of MCT4 which has been shown to act as a “resistance factor” to MCT1 inhibition [38, 39]. Therefore, to verify the ability of AZD3965 to inhibit MCT1 transporter activity, we treated Raji B lymphocytes, which express MCT1 but not MCT4 [41], with the same concentrations of AZD3965 that we used with DU145 cells. We found that AZD3965 inhibited ^14^C-lactic acid uptake by approximately 90% at 1 μM in Raji cells (Figure S2B), demonstrating that AZD3965 is able to target MCT1-mediated uptake of ^14^C-lactic acid, even though overall uptake may appear unchanged in the presence of MCT4. Using comparable concentrations of AZD3965, we found that up to 10 μM AZD3965 did not affect EGF- or HGF-induced cell scattering (Figure 5D) or wound healing (Figure 5E) in DU145 cells. Also, AZD3965 did not affect EGF- or HGF-induced signaling or c-Met expression (Figure 5F). Based on these data, we conclude that MCT1 expression, but not MCT1 activity, is necessary for EGF- and HGF-induced cell motility and c-Met activation and expression.

## DISCUSSION

MCT1 expression has been correlated with decreased survival and advanced stages of progression in cancer patients [24–26]. Specifically, the transporter activity of MCT1 has been demonstrated to contribute to tumor progression [24, 28, 29]. The majority of these studies have used broad inhibitors targeting multiple MCTs to examine the role of MCT1 in tumor progression. Here, we used both general and specific MCT1 inhibitors, in addition to genetic approaches to challenge our hypothesis. Our findings, demonstrating that reduced expression of MCT1 dramatically limits EGF- and HGF-induced cell motility, are somewhat expected given the known role of MCT1 transporter activity in tumor progression. However, our data suggesting that MCT1 regulates tumor cell motility and HGF/c-Met signaling *independent of transporter activity* is novel in that it begins to establish a previously undefined function of MCT1.

The vast majority of available data on the connection between MCT1 and cancer focuses on the contribution of MCT1 transporter activity to tumor progression. The data presented herein implicate that MCT1 has an additional, non-transporter role. Notably, there is minimal evidence of transporters exhibiting tumor-promoting activities aside from their primary transporting function. One study found that a sodium iodide transporter serves to regulate tumor cell motility and invasion independent of its transporter activity by interacting with a guanine nucleotide exchange factor [42]. It is possible that MCT1 may act in a similar manner; however, this remains to be investigated.

The importance of the data presented herein can be applied to discussions of efficacy regarding clinical MCT1 inhibitors. For example, AZD3965 is a promising MCT1 inhibitor that is currently in clinical trials (ClinicalTrials.gov identifier: NCT01791595). These data suggest that merely targeting transporter activity may not be enough to reduce tumor progression, specifically in tumors that exploit HGF/c-Met for tumor cell progression, such as Ras-mutated cancers [43–45], notably, some of the most common mutations in cancer [46]. Therefore, targeting MCT1 expression, which would concurrently target transporter activity, may prove to be more beneficial than targeting transporter activity alone.

In conclusion, it is known that molecular factors in and surrounding a tumor can influence the progression of cancer to a lethal phenotype. Particularly, HGF signaling through its cognate receptor, c-Met, can induce cancer cells to undergo an EMT typified by loss of cell-cell adhesions and increased cell motility, leading to tumor cell invasion and metastasis; the same is true for EGF signaling [47]. These invasive and metastatic tumors are the leading cause of all cancer-related deaths. We provide evidence that EGF- and HGF-induced phenotypic changes, ultimately leading to cancer-related death, are dependent on MCT1 expression, independent of MCT1 transporter activity. Specifically, our data suggest that MCT1 may regulate HGF-mediated effects at least partially through down-regulation of the c-Met receptor, a focus of future studies. However, the influence of MCT1 on EGF-mediated motility in the absence of an effect on EGFR expression or activation highlights a more general mechanism of MCT1 regulation of growth factor-stimulated tumor cell motility. A better understanding of these processes will, hopefully, lead to novel therapeutic approaches to slow progressive changes in tumor cells.

## MATERIALS AND METHODS

### Cell culture

All cells were purchased from American Type Culture Collection and grown at 37^°^C with 5% CO_2_. DU145 prostate cancer, HCC1806 breast cancer, and Raji B cell lymphoma cells were grown in RPMI 1640 (Cellgro; Manassas, VA) containing 10% fetal bovine serum (Gemini; West Sacramento, CA) and penicillin/streptomycin (Cellgro). Stable knockdown cell lines were maintained in complete media with 1.8 μg/ml puromycin.

### Materials

Lactic acid, methyl-lactic acid and pyruvic acid were purchased from Sigma-Aldrich (St. Louis, MO). AZD3965 was a gift from AstraZeneca (Macclesfield, England, UK).

### Reverse transcription PCR

DU145 and HCC1806 cells were grown to approximately 70% confluency. Cell pellets were collected and homogenized in Trizol (Invitrogen; Carlsbad, CA). Total RNA was isolated according to the manufacturer protocol. First Strand cDNA synthesis kit (Invitrogen) was used to make cDNA. For endpoint PCR analysis, DU145 cDNA was subjected to 40 cycles. Primers were designed and purchased through Integrated DNA Technologies (Coralville, IA). Primers sequences are shown in Table 1. Resulting PCR products were run on a 2% agarose gel.

**Table 1:** Primers used for endpoint PCR analysis

Gene	Forward (5ʹ → 3ʹ)	Reverse (5ʹ → 3ʹ)
MCT1	GGAGGGAATGATTGGTAGCAAAGG	TGCCAGAAATCTACTGATGCTAGG
MCT2	ACTGATGAGACTTCCTGCCTT	TGCCTCTGTATTCAGGAGTGATTC
MCT3	GCCTGGTGGATGTGTTGAAGAACT	TCGCCTCTATTTCTGGTTCTGTGG
MCT4	TGCTCTTCGGCTGTTTCGTCATCA	CTGGAAGTTGAGTGCCAAACCCAA
sMCT1	GTGGCTGGATTTGCATCCGTGATT	ATCTCTGCACCTGGGATTGGTTGA
GAPDH	CATGTTCGTCATGGGTGTGAACCA	CCTGCTTCACCACCTTCTTGATGT

### Western blot analysis

DU145 and HCC1806 cells were seeded to approximately 70% confluency. Cells were serum-starved 30 minutes and then treated with 33 ng/ml HGF (EMD Millipore; Billerica, MA) or 100 ng/ml EGF (Sigma-Aldrich) for 20 minutes in serum-free RPMI 1640. Cells were lysed in boiling Laemmli (125 mM Tris, 4% SDS, 0.01% bromophenol blue, 30% sucrose) containing 0.5% β-mercaptoethanol and boiled for approximately 7 minutes. Following at least one freeze-thaw cycle, equal volumes of protein were loaded into polyacrylamide gels and normalized to load controls. Primary antibodies used were phospho-Met (Y1234/Y1235) and phospho-EGFR (S845) and phospho-Akt (S473) (Cell Signaling Technology; Beverly, MA), MCT1 (H-70) and MCT4 (H-90) and EGFR (1005) (Santa Cruz Biotechnology; Santa Cruz, CA), c-Met (C-28) (Life Technologies; Grand Island, NY), CD147 (R&D Systems; Minneapolis, MN). α-actin (Sigma-Aldrich) and β-tubulin (Neomarkers, Inc.; Fremont, CA) were used as load controls. Secondary antibodies used were horseradish peroxidase conjugated anti-rabbit and anti-mouse (GE Healthcare; Pittsburgh, PA). ECL 2 was used for detection (Thermo Scientific; Rockford, IL).

### RNA interference

Mission Lentiviral Transduction Particles (Sigma-Aldrich) were used to generate DU145 and HCC1806 cells with stable knockdown of MCT1 and stable DU145 MCT4 KD cells. Cells were plated at approximately 30% confluency then treated with 10 μl of lentivirus and 8 μg/ml polybrene. After 24 hours, media was changed to 10% FBS RPMI for 24 hours prior to selecting in 10% FBS RPMI containing 1.8 μg/ml puromycin. For both MCT1 KD and MCT4 KD, five distinct shRNA sequences were used to establish five different cell lines, including a non-targeted shRNA control.

### Scattering assay

Cells were sparsely seeded and grown to approximately 60% confluency in serum-containing media. Cells were treated overnight in serum-free media with or without various inhibitors and growth factors. All conditions were used at pH 7.2–7.4. Following overnight treatment, cells were fixed with cold 4% paraformaldehyde at room temperature for 20 minutes, washed twice with 1X PBS, permeablized with 0.1% Triton X-100 for 15 minutes, washed twice with 1X PBS, and stained with Oregon Green 488 phalloidin (Invitrogen) diluted 1:200 in 1X PBS for 30 minutes. Representative images of three independent experiments are shown. Images were acquired using an Eclipse TE300 inverted microscope (Nikon; Tokyo, Japan) and are shown at 10X magnification.

### Wound healing and invasion assay

Cells were grown to approximately 100% confluency in 96-well ImageLock^™^ plates (Essen Bioscience; Ann Arbor, MI). Confluent monolayers were wounded using the WoundMaker^™^ (Essen Bioscience) and then washed once with serum-containing media to remove any loose cell debris. For invasion, a 1:5 dilution of Matrigel^®^ in serum-free RPMI was laid on top of the wounded monolayer and allowed to solidify 15 minutes at 37°C prior to treatment. Cells were treated with various growth factors or inhibitors in serum-free media prior to incubation within an IncuCyte^™^ ZOOM imaging system (Essen Bioscience) housed within an incubator maintained at 37^°^C with 5% CO_2_ for the indicated times. The IncuCyte^™^ ZOOM acquired 10× images every 4 hours and collected quantitative information that was graphed using Microsoft Excel. Data are representative of three independent experiments performed with quadruplicate replicates.

### Lactic acid export assay

Lactic acid export was measured following overnight treatment of approximately 70% confluent cells in serum-free conditions. Cell supernatant was collected and assayed using a lactic acid measurement kit (Pointe Scientific; Canton, MI). Following removal of supernatant, cell lysates were collected using Pierce lysis buffer (Tris 25 mM, NaCl 150 mM, EDTA 1 mM, NP-40 1%, Glycerol 5%, pH 7.4) and lactic acid production was normalized to protein. Protein measurements were obtained using Pierce 660 nm protein assay reagent (Thermo Scientific). Data are shown as percent lactic acid export compared to non-target cells of which export was set at 100%. The data are representative of five independent experiments.

### Lactic acid uptake assay

DU145 cells were seeded and allowed to grow to approximately 70% confluency in 10% FBS RPMI. Immediately prior to treatment, cells were washed twice with room temperature HEPES buffer. For the timecourse, HEPES containing 0.2 μCi/mL (1.34 μM) ^14^C-lactic acid (American Radiolabeled Chemicals; St. Louis, MO) was added to the cells for either 1, 2, or 4 hours at 37^°^C. For the competition and inhibitor assays, cells were treated with or without 40 mM unlabeled lactic acid, 40 mM unlabeled pyruvic acid, 40 μM phloretin, or various concentrations of AZD3965 immediately prior to spiking in 1.34 μM ^14^C-lactic acid for 1 hour. Radiolabeled cells were also incubated at 4^°^C for the indicated times in parallel to control for radioisotope binding to the cell surface and resulting readings were subtracted out of final measurements. For all experiments, uptake was stopped by washing with cold HEPES four times. Cold Pierce lysis buffer was used to collect cell lysates. Lysates were spun end-over-end at 4^°^C for 30 minutes to help break up the membrane. Lysates were then centrifuged at 4^°^C for 5 minutes at 13 000 rpm. Supernatants were transferred to clean microcentrifuge tubes. Two-thirds of the supernatant was used to measure counts per minute (CPM) using a Beckman LS 6500 scintillation system. Pierce 660 nm protein assays were also performed for each sample. Timecourse results are shown as mean CPM/mg protein over time. All other data are presented as the percent uptake normalized to protein and compared to baseline ^14^C-lactic acid uptake set at 100%. The data shown are representative of three independent experiments.

### ATP assay

DU145 NT, MCT1 KD, and MCT4 KD cells were grown to approximately 60% confluency in 96-well plates. Complete media was removed from the cells and replaced with serum-free media. After 24 hours at 37^°^C, levels of ATP were measured using the Cell Titer Glo kit (Promega; Madison, WI). The data represent the results from three independent experiments performed with 12 replicates per cell line.

### Statistical analysis and data presentation

All quantitative data are shown as mean ± SEM. ANOVA was used to determine differences between groups followed by Tukey's multiple comparison test where appropriate. Area under a curve (AUC) was estimated by using trapezoidal rule for individual cell lines along time. ANOVA was used to compare the AUC among cell lines. Student's *t*-test was used to compare the means between treatments or cell lines for bar graphs. SAS 9.4 statistical software (SAS Inc.; Gary, NC) was used for all statistical data analyses. All statistically significant differences are annotated within the figures. Differences were considered statistically significant at *p* < 0.05.

## SUPPLEMENTARY MATERIALS FIGURES


