# Supplementary material for: Monocarboxylate transporter 1 contributes to growth factor-induced tumor cell migration independent of transporter activity
Source: Oncotarget. 2016 Apr 26;7(22):32695–706. doi: 10.18632/oncotarget.9016 (PMC5078044; doi:10.18632/oncotarget.9016)
Supplement: Supplementary file 1 [file oncotarget-07-32695-s001.pdf]

Monocarboxylate transporter 1 contributes to growth factor-induced tumor cell migration independent of transporter activity

Supplementary Materials

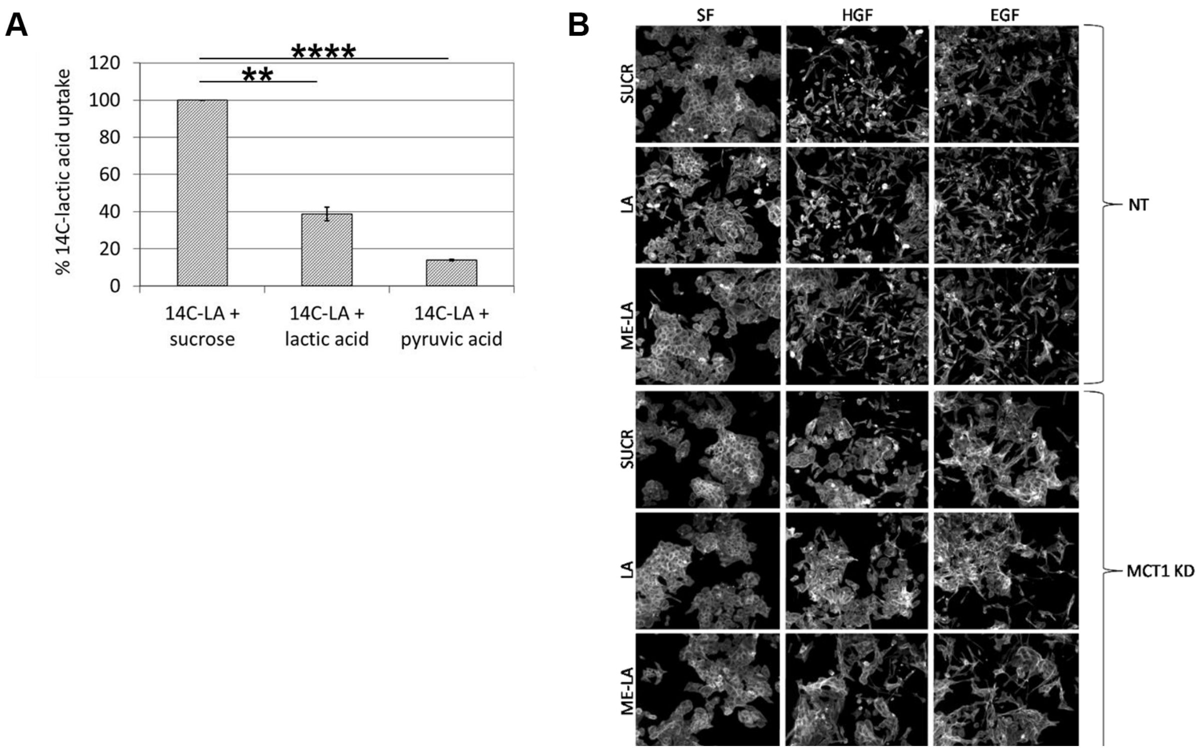

Supplementary Figure S1: The substrates of MCT1 are not sufficient to rescue HGF- and EGF-induced cell motility in MCT1 KD cells. (**A**) DU145 cells were incubated for 1 hour in HEPES buffer containing 0.2 µCi/mL (1.34 µM) ^{14}C-lactic acid with 40 mM sucrose, 40 mM lactic acid, or 40 mM pyruvic acid. Data are shown as percent ^{14}C-lactic acid uptake normalized to control set at 100%; $n = 3$. (**B**) DU145 NT and MCT1 KD cells were treated in serum-free (SF) media with 40 mM sucrose (SUCR), 40 mM lactic acid (LA), or 40 mM methyl-lactic acid (ME-LA) with or without 33 ng/ml HGF or 100 ng/ml EGF overnight. Cells were fixed and stained for actin. Representative 10× images are shown. Quantitative data represent mean ± SEM. $**p < 0.01$, $****p < 0.0001$.

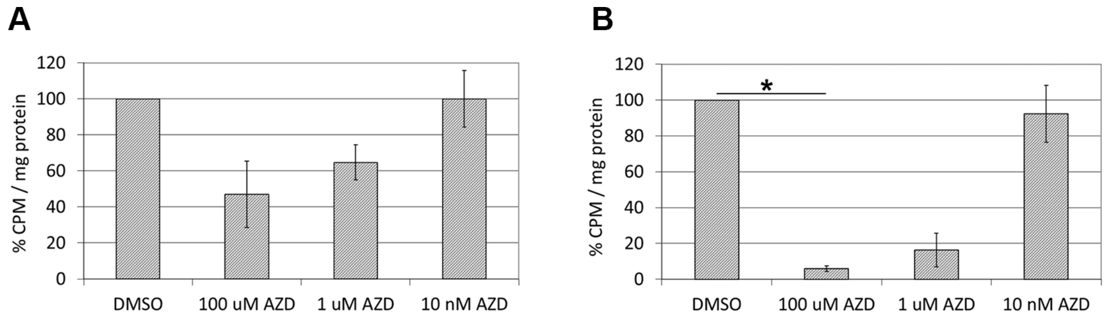

Supplementary Figure S2: AZD3965-mediated inhibition of lactic acid uptake through MCT1 is partially obscured in cells expressing MCT4. (**A**) DU145 ($MCT1^{+}$, $MCT4^{+}$) and (**B**) Raji ($MCT1^{+}$, $MCT4^{-}$) cells were treated with 100 µM, 1 µM, or 10 nM AZD3965 in HEPES buffer containing ^{14}C-lactic acid for the indicated times; $n = 3$. $*p < 0.05$.
